# Differences in genomic patterns and clinical outcomes between African-American and White patients with myelodysplastic syndromes

**DOI:** 10.1038/bcj.2017.82

**Published:** 2017-09-01

**Authors:** A Nazha, K Al-Issa, B Przychodzen, N Abuhadra, C Hirsch, J P Maciejewski, M A Sekeres

**Affiliations:** 1 Department of Translation Hematology and Oncology Research, Leukemia Program, Cleveland Clinic, Taussig Cancer Institute, Cleveland, OH, USA

African-American (AA) patients have a younger age at diagnosis and worse outcomes compared to Whites (WTs) across many cancers, including acute myeloid and acute lymphoblastic leukemias.^1, 2, 3^ Although inferior outcomes for AAs compared to WTs may be related to differential access to medical care or socioeconomic status, disease biology and genetic fingerprint may have a role. A recent study from the Cancer Genome Atlas has shown that WT and AA women with stage I to III breast cancer have differences in the genomic landscape that is correlated with a higher incidence of *TP53* mutations, fewer PIK3CA mutations and greater intratumor genetic heterogeneity for AA compared to WTs. This finding suggests that the poor outcome of AA women with breast cancer is not only driven by the disease characteristics such as younger age at diagnosis and a higher incidence of triple-negative disease, but also related to differences in the genomic landscape of their disease.

The incidence rate of myelodysplastic syndromes (MDS) and age at diagnosis in national cancer registries for AAs is lower than that for WTs,^4^ though detailed biological and clinical characteristics and outcomes of AA patients with MDS compared to WTs have not been reported. The aim of this study is to define the clinical characteristics, outcomes and genomic landscape of AA MDS patients compared to WTs.

We collected mutational and clinical data on MDS patients diagnosed at our institution between January 2000 and January 2012. Next-generation gene-targeted deep sequencing of 60 commonly mutated genes in myeloid malignancies were analyzed as individual mutations and then grouped into several functional pathways. Details of sequencing methods and the region of the targeted genes is summarized in Supplementary Materials. International prognostic scoring system-revised (IPSS-R) scores were calculated as described previously.^5^ Overall survival (OS) was measured from the time of diagnosis to time of death or last follow-up. Time-to-event analyses were performed by the Kaplan–Meier method, with curves compared by log-rank test. Differences among variables were evaluated by the Fisher’s exact test and Mann–Whitney *U* test for categorical and continuous variables, respectively.

Of 341 patients, 44 (13%) were AA. Comparing WTs to AAs, patients had a similar median age 68 (range, 24–93) vs 68 (range, 20–87) years, *P*=0.64, absolute neutrophil count 1.6 (range, 0.02–170) vs 2.23 (range, 0.39–31.47) × 10^9^/l, *P*=0.11, hemoglobin 9.7 (range, 3.9–14.6) vs 9.4 (range, 6.2–14.2) g/dl, *P*=0.06, platelets 93 (range, 9–776) vs 91 (range, 4–681) × 10^9^/l, *P*=0.64 and bone marrow blast percentage 2% (range, 0–19) vs 3% (range, 0–19), *P*=0.22, respectively. IPSS-R risk category distribution for WTs and AA was: very low 15 vs 9%, low 35 vs 30%, intermediate 18 vs 18%, high 16 vs 23%, very high 10 vs 18% and not applicable 6 vs 2%, respectively. Among AA patients, 25% had very-poor-risk cytogenetics (complex >3) compared to 10% of WTs (*P*=0.008), which led to 41% of AA patients having high- and very-high-risk IPSS-R scores compared to 26% of WTs (*P*=0.035). Further, WTs were more likely to receive any treatment (86 vs 66%, *P*<0.001) and allogeneic hematopoietic cell transplantation (HCT) (15 vs 5%, *P*=0.04) compared to AAs; however, acute myeloid leukemia transformation rates were similar (21 vs 25%, *P*=0.31, respectively). With a median follow-up of 36 months (range, 0.9–128.5), the median OS for AAs was 17.9 vs 27.5 months for WTs (*P*=0.03, Figure 1). In a multivariable Cox analysis that included age and IPSS-R, AA patients retained their worse outcome compared to WTs (hazard ratio 1.68, confidence interval 1.17–2.41, *P*=0.005).

Somatic mutational data were available for 321 patients. Overall, the most frequently mutated genes were: *TET2* (16%), *SF3B1* (13%), *ASXL1* (13%), *DNMT3A* (10%), *BCOR/BCLOR1* (10%), *STAG2* (10%), *U2AF1* (8%), *ZRSR2* (7%) and *TP53* (5%) (mutation distribution, Figure 2).

AA patients were more likely to have *TP53* (17 vs 4%, *P*=0.04) and *ZRSR2* mutations (21 vs 6%, *P*=0.02). As a group, mutations in transcription factors and chromatin modification were more common in WTs compared to AA patients (*P*=0.02 and 0.049, respectively; Figure 2).

In this study, we have shown that AA patients with MDS had worse OS compared to WTs even after adjustment for clinical variables and age. We also demonstrated that AA patients are more likely to have poor-risk cytogenetics and high-/very-high-risk categories per IPSS-R, and a higher incidence of poor-risk mutations such as *TP53*. To our knowledge, this is the first report to show inferior outcome of AA patients with MDS, and correlate this outcome with disease characteristics and genomic fingerprint.

Further, AA patients were less likely to receive any treatment, including HCT, suggesting a potential impact of sociodemographic factors or difficulty identifying a suitable HCT donor on their outcome as well.

Although our patient cohort of AA is relatively small, this observation is important as it highlights that disparities in cancer in general are related both to access to care, and to disease biology and genomic characteristics. Further, these findings support more research to investigate its relevance in larger patient cohorts and whether this biologic differences translate to differential therapy responsiveness.

## Figures and Tables

**Figure 1:**
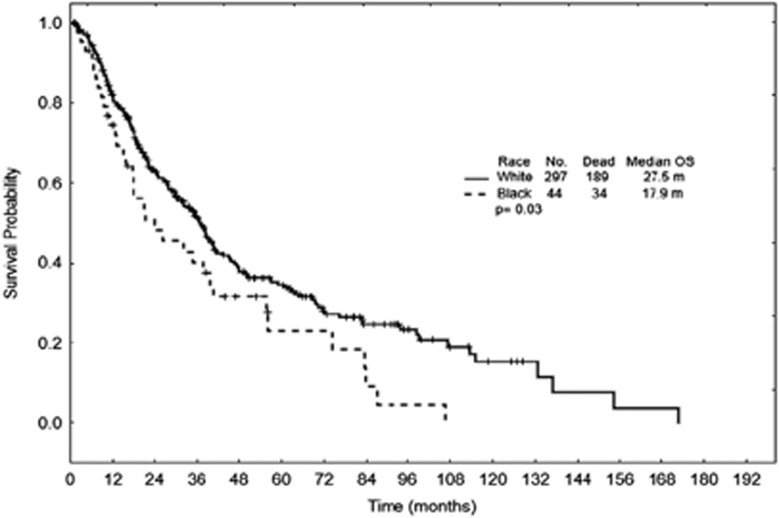
Overall survival by race.

**Figure 2:**
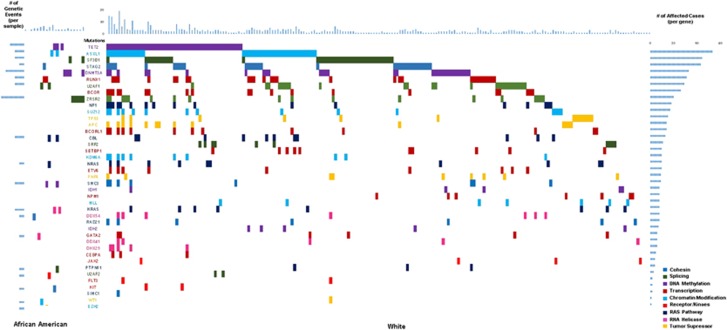
Mutation distribution in African-American patients versus White.
