# Supplementary material for: Differences in genomic patterns and clinical outcomes between African-American and White patients with myelodysplastic syndromes
Source: Blood Cancer J. 2017 Sep 1;7(9):e602–. doi: 10.1038/bcj.2017.82 (PMC5709751; doi:10.1038/bcj.2017.82)
Supplement: Supplementary Material [file bcj201782x1.docx]

***Sample Processing, DNA Sequencing and Mutational Analysis***

Genomic DNA was extracted from peripheral blood and bone marrow mononuclear cells per manufacturer’s protocol. Direct sequencing was performed on coding exons of 60 genes (Supplementary Data) using the Illumina TrueSeq Custom Amplicon kit (San Diego, CA, USA). The mean sequencing coverage for the targeted regions was 650 ×. For germline confirmation, mutations were analyzed in non-clonal CD3þ cells whenever DNA was available. Bidirectional sequencing was performed by standard techniques using an ABI 3730xl DNA analyser (Applied Biosystems, Foster City, CA, USA). Putative variants were extracted using GATK3.3 pipeline, following recommended best practices for variant discovery. Variants with at least 10 positive reads and a variant allele frequency of 5% were prioritized for further processing and annotation. VCF files generated were used as an input for Annovar and were annotated with multiple databases (dbSNP138, COSMIC, ExacDb). Variants found in ExacDb with allelic frequency 40.0001 were excluded.

**Table 1:** Selected target regions of recurrently mutated genes (60) included in the study

| **Target** | **Chromosome** | **Start Position** | **End Position** |
| --- | --- | --- | --- |
| *APC* | chr5 | 112043079 | 112043314 |
| *APC* | chr5 | 112043249 | 112043488 |
| *APC* | chr5 | 112043441 | 112043699 |
| *APC* | chr5 | 112073527 | 112073799 |
| *APC* | chr5 | 112074023 | 112074265 |
| *APC* | chr5 | 112090535 | 112090793 |
| *APC* | chr5 | 112101954 | 112102192 |
| *APC* | chr5 | 112102757 | 112102993 |
| *APC* | chr5 | 112102943 | 112103199 |
| *APC* | chr5 | 112111277 | 112111517 |
| *APC* | chr5 | 112116426 | 112116665 |
| *APC* | chr5 | 112128064 | 112128294 |
| *APC* | chr5 | 112136947 | 112137211 |
| *APC* | chr5 | 112151121 | 112151357 |
| *APC* | chr5 | 112154568 | 112154799 |
| *APC* | chr5 | 112154744 | 112154973 |
| *APC* | chr5 | 112154922 | 112155176 |
| *APC* | chr5 | 112157554 | 112157818 |
| *APC* | chr5 | 112162774 | 112163003 |
| *APC* | chr5 | 112164426 | 112164661 |
| *APC* | chr5 | 112164608 | 112164852 |
| *APC* | chr5 | 112170523 | 112170754 |
| *APC* | chr5 | 112170699 | 112170932 |
| *APC* | chr5 | 112173141 | 112173366 |
| *APC* | chr5 | 112173287 | 112173516 |
| *APC* | chr5 | 112173429 | 112173654 |
| *APC* | chr5 | 112173573 | 112173797 |
| *APC* | chr5 | 112173713 | 112173940 |
| *APC* | chr5 | 112173861 | 112174085 |
| *APC* | chr5 | 112174001 | 112174225 |
| *APC* | chr5 | 112174141 | 112174367 |
| *APC* | chr5 | 112174283 | 112174507 |
| *APC* | chr5 | 112174425 | 112174649 |
| *APC* | chr5 | 112174577 | 112174806 |
| *APC* | chr5 | 112174721 | 112174945 |
| *APC* | chr5 | 112174863 | 112175087 |
| *APC* | chr5 | 112175005 | 112175230 |
| *APC* | chr5 | 112175145 | 112175370 |
| *APC* | chr5 | 112175285 | 112175510 |
| *APC* | chr5 | 112175431 | 112175655 |
| *APC* | chr5 | 112175575 | 112175799 |
| *APC* | chr5 | 112175717 | 112175941 |
| *APC* | chr5 | 112175865 | 112176090 |
| *APC* | chr5 | 112176011 | 112176236 |
| *APC* | chr5 | 112176157 | 112176381 |
| *APC* | chr5 | 112176301 | 112176526 |
| *APC* | chr5 | 112176473 | 112176710 |
| *APC* | chr5 | 112176621 | 112176846 |
| *APC* | chr5 | 112176761 | 112176985 |
| *APC* | chr5 | 112176905 | 112177130 |
| *APC* | chr5 | 112177049 | 112177274 |
| *APC* | chr5 | 112177193 | 112177417 |
| *APC* | chr5 | 112177335 | 112177559 |
| *APC* | chr5 | 112177473 | 112177697 |
| *APC* | chr5 | 112177621 | 112177846 |
| *APC* | chr5 | 112177759 | 112177984 |
| *APC* | chr5 | 112177913 | 112178141 |
| *APC* | chr5 | 112178059 | 112178284 |
| *APC* | chr5 | 112178209 | 112178433 |
| *APC* | chr5 | 112178355 | 112178579 |
| *APC* | chr5 | 112178491 | 112178717 |
| *APC* | chr5 | 112178633 | 112178858 |
| *APC* | chr5 | 112178777 | 112179003 |
| *APC* | chr5 | 112178923 | 112179147 |
| *APC* | chr5 | 112179063 | 112179290 |
| *APC* | chr5 | 112179211 | 112179436 |
| *APC* | chr5 | 112179387 | 112179611 |
| *APC* | chr5 | 112179555 | 112179779 |
| *APC* | chr5 | 112179723 | 112179948 |
| *APC* | chr5 | 112179867 | 112180103 |
| *APC* | chr5 | 112180045 | 112180319 |
| *APC* | chr5 | 112180263 | 112180537 |
| *APC* | chr5 | 112180461 | 112180686 |
| *APC* | chr5 | 112180619 | 112180846 |
| *APC* | chr5 | 112180785 | 112181031 |
| *APC* | chr5 | 112180973 | 112181233 |
| *APC* | chr5 | 112181179 | 112181407 |
| *APC* | chr5 | 112181343 | 112181572 |
| *APC* | chr5 | 112181489 | 112181744 |
| *APC* | chr5 | 112181675 | 112181925 |
| *APC* | chr5 | 112181869 | 112182118 |
| *ASXL1* | chr20 | 30946024 | 30946252 |
| *ASXL1* | chr20 | 30946180 | 30946454 |
| *ASXL1* | chr20 | 30946392 | 30946623 |
| *ASXL1* | chr20 | 30946574 | 30946831 |
| *ASXL1* | chr20 | 30954154 | 30954402 |
| *ASXL1* | chr20 | 30956786 | 30957028 |
| *ASXL1* | chr20 | 30959840 | 30960065 |
| *ASXL1* | chr20 | 30960010 | 30960234 |
| *ASXL1* | chr20 | 31015802 | 31016027 |
| *ASXL1* | chr20 | 31015966 | 31016192 |
| *ASXL1* | chr20 | 31016140 | 31016379 |
| *ASXL1* | chr20 | 31017114 | 31017370 |
| *ASXL1* | chr20 | 31017675 | 31017949 |
| *ASXL1* | chr20 | 31019007 | 31019233 |
| *ASXL1* | chr20 | 31019183 | 31019421 |
| *ASXL1* | chr20 | 31019375 | 31019602 |
| *ASXL1* | chr20 | 31020652 | 31020912 |
| *ASXL1* | chr20 | 31020960 | 31021186 |
| *ASXL1* | chr20 | 31021134 | 31021365 |
| *ASXL1* | chr20 | 31021310 | 31021536 |
| *ASXL1* | chr20 | 31021486 | 31021751 |
| *ASXL1* | chr20 | 31022110 | 31022337 |
| *ASXL1* | chr20 | 31022260 | 31022485 |
| *ASXL1* | chr20 | 31022410 | 31022634 |
| *ASXL1* | chr20 | 31022550 | 31022777 |
| *ASXL1* | chr20 | 31022696 | 31022920 |
| *ASXL1* | chr20 | 31022842 | 31023066 |
| *ASXL1* | chr20 | 31022982 | 31023206 |
| *ASXL1* | chr20 | 31023126 | 31023350 |
| *ASXL1* | chr20 | 31023268 | 31023492 |
| *ASXL1* | chr20 | 31023412 | 31023636 |
| *ASXL1* | chr20 | 31023558 | 31023783 |
| *ASXL1* | chr20 | 31023706 | 31023931 |
| *ASXL1* | chr20 | 31023852 | 31024076 |
| *ASXL1* | chr20 | 31023996 | 31024220 |
| *ASXL1* | chr20 | 31024140 | 31024365 |
| *ASXL1* | chr20 | 31024290 | 31024514 |
| *ASXL1* | chr20 | 31024434 | 31024660 |
| *ASXL1* | chr20 | 31024580 | 31024804 |
| *ASXL1* | chr20 | 31024726 | 31024951 |
| *ASXL1* | chr20 | 31024868 | 31025092 |
| *ASXL1* | chr20 | 31025022 | 31025249 |
| *ASXL1* | chr20 | 31025174 | 31025398 |
| *ASXL1* | chr20 | 31025312 | 31025537 |
| *ASXL1* | chr20 | 31025454 | 31025678 |
| *ASXL1* | chr20 | 31025596 | 31025822 |
| *ASXL1* | chr20 | 31025752 | 31025978 |
| *ASXL1* | chr20 | 31025926 | 31026172 |
| *ASXL1* | chr20 | 31026116 | 31026343 |
| *ASXL1* | chr20 | 31026298 | 31026522 |
| *ASXL1* | chr20 | 31026472 | 31026696 |
| *ASXL1* | chr20 | 31026642 | 31026875 |
| *ASXL1* | chr20 | 31026822 | 31027093 |
| *ASXL1* | chr20 | 31027042 | 31027301 |
| *BCOR* | chrY | 21635844 | 21636073 |
| *BCOR* | chrY | 21644557 | 21644778 |
| *BCOR* | chrY | 21645569 | 21645794 |
| *BCOR* | chrX | 39910376 | 39910619 |
| *BCOR* | chrX | 39910558 | 39910782 |
| *BCOR* | chrX | 39910710 | 39910943 |
| *BCOR* | chrX | 39910886 | 39911134 |
| *BCOR* | chrX | 39911078 | 39911320 |
| *BCOR* | chrX | 39911260 | 39911492 |
| *BCOR* | chrX | 39911438 | 39911703 |
| *BCOR* | chrX | 39913014 | 39913238 |
| *BCOR* | chrX | 39913180 | 39913408 |
| *BCOR* | chrX | 39913354 | 39913590 |
| *BCOR* | chrX | 39914590 | 39914848 |
| *BCOR* | chrX | 39916381 | 39916629 |
| *BCOR* | chrX | 39921271 | 39921519 |
| *BCOR* | chrX | 39921471 | 39921745 |
| *BCOR* | chrX | 39921878 | 39922102 |
| *BCOR* | chrX | 39922044 | 39922289 |
| *BCOR* | chrX | 39922734 | 39922964 |
| *BCOR* | chrX | 39922914 | 39923146 |
| *BCOR* | chrX | 39923092 | 39923337 |
| *BCOR* | chrX | 39923462 | 39923688 |
| *BCOR* | chrX | 39923636 | 39923906 |
| *BCOR* | chrX | 39930199 | 39930438 |
| *BCOR* | chrX | 39930823 | 39931055 |
| *BCOR* | chrX | 39931477 | 39931704 |
| *BCOR* | chrX | 39931623 | 39931847 |
| *BCOR* | chrX | 39931767 | 39931992 |
| *BCOR* | chrX | 39931917 | 39932142 |
| *BCOR* | chrX | 39932065 | 39932290 |
| *BCOR* | chrX | 39932211 | 39932435 |
| *BCOR* | chrX | 39932357 | 39932582 |
| *BCOR* | chrX | 39932497 | 39932723 |
| *BCOR* | chrX | 39932645 | 39932870 |
| *BCOR* | chrX | 39932791 | 39933016 |
| *BCOR* | chrX | 39932935 | 39933160 |
| *BCOR* | chrX | 39933077 | 39933301 |
| *BCOR* | chrX | 39933217 | 39933442 |
| *BCOR* | chrX | 39933519 | 39933746 |
| *BCOR* | chrX | 39933679 | 39933905 |
| *BCOR* | chrX | 39933859 | 39934084 |
| *BCOR* | chrX | 39934035 | 39934268 |
| *BCOR* | chrX | 39934215 | 39934474 |
| *BCOR* | chrX | 39935676 | 39935939 |
| *BCOR* | chrX | 39937070 | 39937312 |
| *BCOR* | chrX | 39956359 | 39956628 |
| *BCOR* | chrX | 39956577 | 39956847 |
| *BCOR* | chrX | 40036147 | 40036419 |
| *BCOR* | chrX | 40036367 | 40036640 |
| *BCORL1* | chrX | 129139133 | 129139373 |
| *BCORL1* | chrX | 129146527 | 129146785 |
| *BCORL1* | chrX | 129146803 | 129147030 |
| *BCORL1* | chrX | 129146965 | 129147191 |
| *BCORL1* | chrX | 129147113 | 129147337 |
| *BCORL1* | chrX | 129147253 | 129147478 |
| *BCORL1* | chrX | 129147397 | 129147622 |
| *BCORL1* | chrX | 129147541 | 129147765 |
| *BCORL1* | chrX | 129147693 | 129147918 |
| *BCORL1* | chrX | 129147837 | 129148061 |
| *BCORL1* | chrX | 129147981 | 129148205 |
| *BCORL1* | chrX | 129148135 | 129148361 |
| *BCORL1* | chrX | 129148283 | 129148507 |
| *BCORL1* | chrX | 129148433 | 129148658 |
| *BCORL1* | chrX | 129148585 | 129148809 |
| *BCORL1* | chrX | 129148727 | 129148952 |
| *BCORL1* | chrX | 129148873 | 129149097 |
| *BCORL1* | chrX | 129149045 | 129149270 |
| *BCORL1* | chrX | 129149215 | 129149441 |
| *BCORL1* | chrX | 129149387 | 129149611 |
| *BCORL1* | chrX | 129149559 | 129149792 |
| *BCORL1* | chrX | 129149745 | 129149970 |
| *BCORL1* | chrX | 129149919 | 129150153 |
| *BCORL1* | chrX | 129150101 | 129150359 |
| *BCORL1* | chrX | 129154933 | 129155204 |
| *BCORL1* | chrX | 129156847 | 129157079 |
| *BCORL1* | chrX | 129158840 | 129159067 |
| *BCORL1* | chrX | 129159010 | 129159236 |
| *BCORL1* | chrX | 129159182 | 129159423 |
| *BCORL1* | chrX | 129162483 | 129162714 |
| *BCORL1* | chrX | 129162663 | 129162935 |
| *BCORL1* | chrX | 129171315 | 129171576 |
| *BCORL1* | chrX | 129173085 | 129173339 |
| *BCORL1* | chrX | 129184665 | 129184933 |
| *BCORL1* | chrX | 129185806 | 129186070 |
| *BCORL1* | chrX | 129189706 | 129189933 |
| *BCORL1* | chrX | 129189860 | 129190092 |
| *BCORL1* | chrX | 129190038 | 129190272 |
| *BCORL1* | chrX | 129190220 | 129190448 |
| *BCORL1* | chrX | 129190396 | 129190628 |
| *BCORL1* | chrX | 129190570 | 129190797 |
| *BCORL1* | chrX | 129190748 | 129190974 |
| *BCORL1* | chrX | 129190920 | 129191154 |
| *BCORL1* | chrX | 129191286 | 129191514 |
| *BCORL1* | chrX | 129191460 | 129191697 |
| *BCORL1* | chrX | 129191642 | 129191916 |
| *BCORL1* | chrX | 129191862 | 129192104 |
| *c5orf25* | chr5 | 175665259 | 175665525 |
| *c5orf25* | chr5 | 175665475 | 175665742 |
| *c5orf25* | chr5 | 175722110 | 175722373 |
| *c5orf25* | chr5 | 175749229 | 175749499 |
| *c5orf25* | chr5 | 175751603 | 175751873 |
| *c5orf25* | chr5 | 175763596 | 175763825 |
| *c5orf25* | chr5 | 175763770 | 175764004 |
| *c5orf25* | chr5 | 175763946 | 175764202 |
| *c5orf25* | chr5 | 175772059 | 175772283 |
| *c5orf25* | chr5 | 175772227 | 175772454 |
| *c5orf25* | chr5 | 175772399 | 175772634 |
| *c5orf25* | chr5 | 175772553 | 175772810 |
| *c5orf25* | chr5 | 175772755 | 175773008 |
| *c5orf25* | chr5 | 175772949 | 175773211 |
| *c5orf25* | chr5 | 177054275 | 177054538 |
| *c5orf25* | chr5 | 177098920 | 177099187 |
| *c5orf25* | chr5 | 177099137 | 177099404 |
| *CBL* | chr11 | 119148794 | 119149030 |
| *CBL* | chr11 | 119148974 | 119149230 |
| *CBL* | chr11 | 119149174 | 119149430 |
| *CEBPA* | chr19 | 33792274 | 33792498 |
| *CEBPA* | chr19 | 33792896 | 33793131 |
| *CEBPA* | chr19 | 33793080 | 33793354 |
| *CSF1R* | chr5 | 149433617 | 149433842 |
| *CSF1R* | chr5 | 149453017 | 149453287 |
| *CUX1* | chr7 | 56978380 | 56978644 |
| *CUX1* | chr7 | 63126368 | 63126632 |
| *cux1* | chr7 | 101459265 | 101459534 |
| *CUX1* | chr7 | 101460851 | 101461123 |
| *CUX1* | chr7 | 101559366 | 101559608 |
| *CUX1* | chr7 | 101671351 | 101671612 |
| *CUX1* | chr7 | 101713588 | 101713856 |
| *CUX1* | chr7 | 101740611 | 101740883 |
| *CUX1* | chr7 | 101747571 | 101747799 |
| *CUX1* | chr7 | 101754951 | 101755197 |
| *CUX1* | chr7 | 101758428 | 101758688 |
| *CUX1* | chr7 | 101801813 | 101802037 |
| *CUX1* | chr7 | 101813697 | 101813965 |
| *cux1* | chr7 | 101821626 | 101821860 |
| *cux1* | chr7 | 101821812 | 101822072 |
| *CUX1* | chr7 | 101833060 | 101833300 |
| *CUX1* | chr7 | 101837093 | 101837325 |
| *CUX1* | chr7 | 101838760 | 101839026 |
| *CUX1* | chr7 | 101839809 | 101840049 |
| *CUX1* | chr7 | 101839993 | 101840236 |
| *CUX1* | chr7 | 101840163 | 101840392 |
| *CUX1* | chr7 | 101840343 | 101840572 |
| *CUX1* | chr7 | 101840523 | 101840768 |
| *CUX1* | chr7 | 101842051 | 101842295 |
| *CUX1* | chr7 | 101843320 | 101843592 |
| *CUX1* | chr7 | 101844515 | 101844739 |
| *CUX1* | chr7 | 101844657 | 101844882 |
| *CUX1* | chr7 | 101844837 | 101845078 |
| *CUX1* | chr7 | 101845025 | 101845259 |
| *CUX1* | chr7 | 101845209 | 101845441 |
| *CUX1* | chr7 | 101845395 | 101845652 |
| *CUX1* | chr7 | 101848357 | 101848597 |
| *CUX1* | chr7 | 101870528 | 101870779 |
| *CUX1* | chr7 | 101870726 | 101870981 |
| *CUX1* | chr7 | 101877207 | 101877446 |
| *CUX1* | chr7 | 101877397 | 101877653 |
| *CUX1* | chr7 | 101882471 | 101882716 |
| *CUX1* | chr7 | 101882671 | 101882926 |
| *CUX1* | chr7 | 101891573 | 101891816 |
| *CUX1* | chr7 | 101891747 | 101892000 |
| *CUX1* | chr7 | 101892491 | 101892717 |
| *CUX1* | chr7 | 101892663 | 101892888 |
| *CUX1* | chr7 | 101892825 | 101893049 |
| *CUX1* | chr7 | 101892997 | 101893239 |
| *CUX1* | chr7 | 101893183 | 101893437 |
| *CUX1* | chr7 | 101916612 | 101916860 |
| *CUX1* | chr7 | 101917490 | 101917744 |
| *CUX1* | chr7 | 101918491 | 101918751 |
| *CUX1* | chr7 | 101921193 | 101921444 |
| *CUX1* | chr7 | 101923298 | 101923529 |
| *CUX1* | chr7 | 101924065 | 101924332 |
| *CUX1* | chr7 | 101925109 | 101925334 |
| *DDX41* | chr5 | 176938453 | 176938680 |
| *DDX41* | chr5 | 176938599 | 176938823 |
| *DDX41* | chr5 | 176938759 | 176938987 |
| *DDX41* | chr5 | 176938939 | 176939163 |
| *DDX41* | chr5 | 176939113 | 176939346 |
| *DDX41* | chr5 | 176939297 | 176939537 |
| *DDX41* | chr5 | 176939483 | 176939710 |
| *DDX41* | chr5 | 176939661 | 176939891 |
| *DDX41* | chr5 | 176939837 | 176940061 |
| *DDX41* | chr5 | 176940015 | 176940239 |
| *DDX41* | chr5 | 176940181 | 176940420 |
| *DDX41* | chr5 | 176940365 | 176940589 |
| *DDX41* | chr5 | 176940537 | 176940766 |
| *DDX41* | chr5 | 176940715 | 176940987 |
| *DDX41* | chr5 | 176941581 | 176941811 |
| *DDX41* | chr5 | 176941737 | 176941961 |
| *DDX41* | chr5 | 176941911 | 176942147 |
| *DDX41* | chr5 | 176942097 | 176942355 |
| *DDX54* | chr12 | 113594875 | 113595099 |
| *DDX54* | chr12 | 113595023 | 113595250 |
| *DDX54* | chr12 | 113595191 | 113595423 |
| *DDX54* | chr12 | 113595375 | 113595613 |
| *DDX54* | chr12 | 113595563 | 113595818 |
| *DDX54* | chr12 | 113595753 | 113596003 |
| *DDX54* | chr12 | 113595955 | 113596196 |
| *DDX54* | chr12 | 113596143 | 113596370 |
| *DDX54* | chr12 | 113596319 | 113596552 |
| *DDX54* | chr12 | 113596501 | 113596749 |
| *DDX54* | chr12 | 113596699 | 113596957 |
| *DDX54* | chr12 | 113599048 | 113599277 |
| *DDX54* | chr12 | 113607546 | 113607770 |
| *DDX54* | chr12 | 113610131 | 113610405 |
| *DDX54* | chr12 | 113612322 | 113612546 |
| *DDX54* | chr12 | 113612490 | 113612721 |
| *DDX54* | chr12 | 113612668 | 113612909 |
| *DDX54* | chr12 | 113612860 | 113613118 |
| *DDX54* | chr12 | 113614522 | 113614746 |
| *DDX54* | chr12 | 113614696 | 113614937 |
| *DDX54* | chr12 | 113616657 | 113616882 |
| *DDX54* | chr12 | 113616821 | 113617064 |
| *DDX54* | chr12 | 113617017 | 113617258 |
| *DDX54* | chr12 | 113617709 | 113617961 |
| *DDX54* | chr12 | 113618705 | 113618959 |
| *DDX54* | chr12 | 113622962 | 113623213 |
| *DDX54* | chr12 | 113623164 | 113623407 |
| *DHX29* | chr5 | 54551976 | 54552248 |
| *DHX29* | chr5 | 54552190 | 54552447 |
| *DHX29* | chr5 | 54555645 | 54555879 |
| *DHX29* | chr5 | 54557137 | 54557404 |
| *DHX29* | chr5 | 54558339 | 54558564 |
| *DHX29* | chr5 | 54558497 | 54558728 |
| *DHX29* | chr5 | 54558677 | 54558907 |
| *DHX29* | chr5 | 54562935 | 54563203 |
| *DHX29* | chr5 | 54563483 | 54563748 |
| *DHX29* | chr5 | 54565162 | 54565395 |
| *DHX29* | chr5 | 54565340 | 54565578 |
| *DHX29* | chr5 | 54566147 | 54566401 |
| *DHX29* | chr5 | 54566345 | 54566601 |
| *DHX29* | chr5 | 54567876 | 54568140 |
| *DHX29* | chr5 | 54569086 | 54569357 |
| *DHX29* | chr5 | 54570315 | 54570539 |
| *DHX29* | chr5 | 54570465 | 54570714 |
| *DHX29* | chr5 | 54570657 | 54570931 |
| *DHX29* | chr5 | 54572047 | 54572315 |
| *DHX29* | chr5 | 54572809 | 54573038 |
| *DHX29* | chr5 | 54572981 | 54573241 |
| *DHX29* | chr5 | 54578902 | 54579127 |
| *DHX29* | chr5 | 54579062 | 54579295 |
| *DHX29* | chr5 | 54579234 | 54579462 |
| *DHX29* | chr5 | 54579404 | 54579646 |
| *DHX29* | chr5 | 54579594 | 54579823 |
| *DHX29* | chr5 | 54581090 | 54581332 |
| *DHX29* | chr5 | 54581481 | 54581751 |
| *DHX29* | chr5 | 54584979 | 54585216 |
| *DHX29* | chr5 | 54585161 | 54585415 |
| *DHX29* | chr5 | 54585989 | 54586262 |
| *DHX29* | chr5 | 54589814 | 54590074 |
| *DHX29* | chr5 | 54591158 | 54591390 |
| *DHX29* | chr5 | 54592003 | 54592235 |
| *DHX29* | chr5 | 54593082 | 54593322 |
| *DHX29* | chr5 | 54603090 | 54603316 |
| *DHX29* | chr5 | 54603246 | 54603481 |
| *DHX29* | chr5 | 54603434 | 54603682 |
| *DHX29* | chr1 | 189960260 | 189960492 |
| *DNMT3A* | chr2 | 25455720 | 25455945 |
| *DNMT3A* | chr2 | 25455862 | 25456089 |
| *DNMT3A* | chr2 | 25456020 | 25456248 |
| *DNMT3A* | chr2 | 25456194 | 25456418 |
| *DNMT3A* | chr2 | 25456360 | 25456587 |
| *DNMT3A* | chr2 | 25456536 | 25456785 |
| *DNMT3A* | chr2 | 25456720 | 25456949 |
| *DNMT3A* | chr2 | 25456882 | 25457123 |
| *DNMT3A* | chr2 | 25457064 | 25457315 |
| *DNMT3A* | chr2 | 25458543 | 25458771 |
| *DNMT3A* | chr2 | 25459778 | 25460034 |
| *DNMT3A* | chr2 | 25461954 | 25462186 |
| *DNMT3A* | chr2 | 25463044 | 25463269 |
| *DNMT3A* | chr2 | 25463216 | 25463447 |
| *DNMT3A* | chr2 | 25463396 | 25463668 |
| *DNMT3A* | chr2 | 25464402 | 25464667 |
| *DNMT3A* | chr2 | 25466638 | 25466862 |
| *DNMT3A* | chr2 | 25466968 | 25467231 |
| *DNMT3A* | chr2 | 25467186 | 25467411 |
| *DNMT3A* | chr2 | 25467364 | 25467638 |
| *DNMT3A* | chr2 | 25468095 | 25468367 |
| *DNMT3A* | chr2 | 25468768 | 25468993 |
| *DNMT3A* | chr2 | 25468948 | 25469182 |
| *DNMT3A* | chr2 | 25469540 | 25469773 |
| *DNMT3A* | chr2 | 25469726 | 25469970 |
| *DNMT3A* | chr2 | 25469922 | 25470183 |
| *DNMT3A* | chr2 | 25470433 | 25470661 |
| *DNMT3A* | chr2 | 25470783 | 25471057 |
| *DNMT3A* | chr2 | 25471007 | 25471261 |
| *DNMT3A* | chr2 | 25472499 | 25472739 |
| *DNMT3A* | chr2 | 25497783 | 25498036 |
| *DNMT3A* | chr2 | 25498336 | 25498562 |
| *DNMT3A* | chr2 | 25504196 | 25504423 |
| *DNMT3A* | chr2 | 25504364 | 25504589 |
| *DNMT3A* | chr2 | 25504532 | 25504773 |
| *DNMT3A* | chr2 | 25504896 | 25505158 |
| *DNMT3A* | chr2 | 25505102 | 25505326 |
| *DNMT3A* | chr2 | 25505272 | 25505516 |
| *DNMT3A* | chr2 | 25505466 | 25505738 |
| *DNMT3A* | chr2 | 25522983 | 25523256 |
| *DNMT3A* | chr2 | 25536677 | 25536951 |
| *DNMT3A* | chr2 | 25536901 | 25537158 |
| *EED* | chr11 | 85955690 | 85955915 |
| *EED* | chr11 | 85956034 | 85956281 |
| *EED* | chr11 | 85956234 | 85956507 |
| *EED* | chr11 | 85961307 | 85961581 |
| *EED* | chr11 | 85963131 | 85963401 |
| *EED* | chr11 | 85966207 | 85966473 |
| *EED* | chr11 | 85967396 | 85967622 |
| *EED* | chr11 | 85968514 | 85968738 |
| *EED* | chr11 | 85975181 | 85975411 |
| *EED* | chr11 | 85977060 | 85977288 |
| *EED* | chr11 | 85979467 | 85979709 |
| *EED* | chr11 | 85987991 | 85988265 |
| *EED* | chr11 | 85988833 | 85989057 |
| *EED* | chr11 | 85988991 | 85989216 |
| *EED* | chr11 | 85989133 | 85989359 |
| *EED* | chr11 | 85989305 | 85989551 |
| *EED* | chr11 | 85989501 | 85989735 |
| *EED* | chr11 | 85989679 | 85989943 |
| *EED* | chrX | 139480427 | 139480657 |
| *ERBB4* | chr2 | 212251830 | 212252054 |
| *ERBB4* | chr2 | 212566727 | 212566957 |
| *ETV6* | chr12 | 11802697 | 11802971 |
| *ETV6* | chr12 | 11802917 | 11803190 |
| *ETV6* | chr12 | 11905355 | 11905626 |
| *ETV6* | chr12 | 11992047 | 11992299 |
| *ETV6* | chr12 | 12006332 | 12006588 |
| *ETV6* | chr12 | 12022233 | 12022461 |
| *ETV6* | chr12 | 12022405 | 12022632 |
| *ETV6* | chr12 | 12022581 | 12022818 |
| *ETV6* | chr12 | 12022765 | 12023022 |
| *ETV6* | chr12 | 12037352 | 12037609 |
| *ETV6* | chr12 | 12038831 | 12039063 |
| *ETV6* | chr12 | 12043748 | 12043972 |
| *ETV6* | chr12 | 12043890 | 12044115 |
| *ETV6* | chr12 | 12044038 | 12044262 |
| *ETV6* | chr12 | 12044200 | 12044430 |
| *ETV6* | chr12 | 12044352 | 12044582 |
| *ETV6* | chr12 | 12044498 | 12044722 |
| *ETV6* | chr12 | 12044658 | 12044889 |
| *ETV6* | chr12 | 12044836 | 12045062 |
| *ETV6* | chr12 | 12045006 | 12045234 |
| *ETV6* | chr12 | 12045182 | 12045406 |
| *ETV6* | chr12 | 12045322 | 12045546 |
| *ETV6* | chr12 | 12045460 | 12045687 |
| *ETV6* | chr12 | 12045622 | 12045846 |
| *ETV6* | chr12 | 12045796 | 12046021 |
| *ETV6* | chr12 | 12045972 | 12046198 |
| *ETV6* | chr12 | 12046292 | 12046527 |
| *ETV6* | chr12 | 12046472 | 12046697 |
| *ETV6* | chr12 | 12046820 | 12047044 |
| *ETV6* | chr12 | 12046958 | 12047184 |
| *ETV6* | chr12 | 12047110 | 12047337 |
| *ETV6* | chr12 | 12047280 | 12047505 |
| *ETV6* | chr12 | 12047442 | 12047672 |
| *ETV6* | chr12 | 12047618 | 12047846 |
| *ETV6* | chr12 | 12047792 | 12048027 |
| *ETV6* | chr12 | 12047970 | 12048195 |
| *ETV6* | chr12 | 12048140 | 12048407 |
| *EZH2* | chr21 | 36972016 | 36972511 |
| *EZH2* | chr7 | 148504346 | 148504573 |
| *EZH2* | chr7 | 148504508 | 148504777 |
| *EZH2* | chr7 | 148504724 | 148504958 |
| *EZH2* | chr7 | 148506038 | 148506263 |
| *EZH2* | chr7 | 148506206 | 148506438 |
| *EZH2* | chr7 | 148506382 | 148506640 |
| *EZH2* | chr7 | 148507398 | 148507652 |
| *EZH2* | chr7 | 148508688 | 148508946 |
| *EZH2* | chr7 | 148511022 | 148511284 |
| *EZH2* | chr7 | 148511975 | 148512227 |
| *EZH2* | chr7 | 148512567 | 148512807 |
| *EZH2* | chr7 | 148513749 | 148514004 |
| *EZH2* | chr7 | 148514279 | 148514523 |
| *EZH2* | chr7 | 148523465 | 148523694 |
| *EZH2* | chr7 | 148523641 | 148523900 |
| *EZH2* | chr7 | 148524225 | 148524454 |
| *EZH2* | chr7 | 148525801 | 148526075 |
| *EZH2* | chr7 | 148526791 | 148527051 |
| *EZH2* | chr7 | 148529643 | 148529905 |
| *EZH2* | chr7 | 148543517 | 148543787 |
| *EZH2* | chr7 | 148544199 | 148544457 |
| *EZH2* | chr7 | 148581205 | 148581447 |
| *FLT3* | chr13 | 28592608 | 28592848 |
| *FLT3* | chr13 | 28602313 | 28602563 |
| *FLT3* | chr13 | 28607985 | 28608214 |
| *FLT3* | chr13 | 28608159 | 28608409 |
| *GATA2* | chr3 | 128198380 | 128198604 |
| *GATA2* | chr3 | 128198544 | 128198776 |
| *GATA2* | chr3 | 128198726 | 128198988 |
| *GATA2* | chr3 | 128198918 | 128199145 |
| *GATA2* | chr3 | 128199098 | 128199339 |
| *GATA2* | chr3 | 128199286 | 128199517 |
| *GATA2* | chr3 | 128199464 | 128199709 |
| *GATA2* | chr3 | 128199818 | 128200058 |
| *GATA2* | chr3 | 128200617 | 128200887 |
| *GATA2* | chr3 | 128202676 | 128202922 |
| *GATA2* | chr3 | 128204445 | 128204674 |
| *GATA2* | chr3 | 128204625 | 128204867 |
| *GATA2* | chr3 | 128204809 | 128205066 |
| *GATA2* | chr3 | 128205017 | 128205273 |
| *GATA2* | chr3 | 128205701 | 128205931 |
| *GATA2* | chr3 | 128206527 | 128206800 |
| *GATA2* | chr3 | 128207168 | 128207402 |
| *GATA2* | chr3 | 128211621 | 128211895 |
| *GATA2* | chr3 | 128211837 | 128212087 |
| *GNB1* | chr1 | 1737867 | 1738103 |
| *GNB1* | chr1 | 1747200 | 1747460 |
| *IDH1* | chr2 | 209113084 | 209113332 |
| *IDH2* | chr15 | 90631901 | 90632143 |
| *IRF4* | chr6 | 391710 | 391970 |
| *IRF4* | chr6 | 392979 | 393223 |
| *IRF4* | chr6 | 393173 | 393421 |
| *IRF4* | chr6 | 394792 | 395046 |
| *IRF4* | chr6 | 395818 | 396066 |
| *IRF4* | chr6 | 397079 | 397352 |
| *IRF4* | chr6 | 398797 | 399033 |
| *IRF4* | chr6 | 401301 | 401526 |
| *IRF4* | chr6 | 401473 | 401712 |
| *IRF4* | chr6 | 401663 | 401918 |
| *IRF4* | chr6 | 404991 | 405253 |
| *IRF4* | chr6 | 407328 | 407552 |
| *IRF4* | chr6 | 407644 | 407868 |
| *IRF4* | chr6 | 407790 | 408017 |
| *IRF4* | chr6 | 407934 | 408158 |
| *IRF4* | chr6 | 408076 | 408302 |
| *IRF4* | chr6 | 408232 | 408457 |
| *IRF4* | chr6 | 408370 | 408594 |
| *IRF4* | chr6 | 408516 | 408742 |
| *IRF4* | chr6 | 408670 | 408895 |
| *IRF4* | chr6 | 408818 | 409043 |
| *IRF4* | chr6 | 408974 | 409205 |
| *IRF4* | chr6 | 409150 | 409375 |
| *IRF4* | chr6 | 409292 | 409516 |
| *IRF4* | chr6 | 409438 | 409662 |
| *IRF4* | chr6 | 409578 | 409802 |
| *IRF4* | chr6 | 409726 | 409951 |
| *IRF4* | chr6 | 409890 | 410114 |
| *IRF4* | chr6 | 410058 | 410301 |
| *IRF4* | chr6 | 410242 | 410466 |
| *IRF4* | chr6 | 410410 | 410635 |
| *IRF4* | chr6 | 410580 | 410804 |
| *IRF4* | chr6 | 410746 | 410971 |
| *IRF4* | chr6 | 410918 | 411150 |
| *IRF4* | chr6 | 411096 | 411322 |
| *IRF4* | chr6 | 411270 | 411512 |
| *JAK2* | chr9 | 5069996 | 5070224 |
| *JAK2* | chr9 | 5073738 | 5074010 |
| *KDM6A* | chrY | 15447784 | 15448008 |
| *KDM6A* | chrX | 44732302 | 44732536 |
| *KDM6A* | chrX | 44732486 | 44732727 |
| *KDM6A* | chrX | 44732668 | 44732901 |
| *KDM6A* | chrX | 44732850 | 44733087 |
| *KDM6A* | chrX | 44820468 | 44820742 |
| *KDM6A* | chrX | 44833862 | 44834104 |
| *KDM6A* | chrX | 44870175 | 44870448 |
| *KDM6A* | chrX | 44879810 | 44880083 |
| *KDM6A* | chrX | 44894147 | 44894385 |
| *KDM6A* | chrX | 44896847 | 44897101 |
| *KDM6A* | chrX | 44910923 | 44911197 |
| *KDM6A* | chrX | 44913003 | 44913227 |
| *KDM6A* | chrX | 44918150 | 44918407 |
| *KDM6A* | chrX | 44918334 | 44918562 |
| *KDM6A* | chrX | 44918508 | 44918772 |
| *KDM6A* | chrX | 44919228 | 44919494 |
| *KDM6A* | chrX | 44920500 | 44920728 |
| *KDM6A* | chrX | 44921821 | 44922073 |
| *KDM6A* | chrX | 44922538 | 44922764 |
| *KDM6A* | chrX | 44922714 | 44922941 |
| *KDM6A* | chrX | 44922884 | 44923147 |
| *KDM6A* | chrX | 44928697 | 44928922 |
| *KDM6A* | chrX | 44928851 | 44929084 |
| *KDM6A* | chrX | 44929031 | 44929261 |
| *KDM6A* | chrX | 44929207 | 44929443 |
| *KDM6A* | chrX | 44929391 | 44929649 |
| *KDM6A* | chrX | 44935895 | 44936129 |
| *KDM6A* | chrX | 44937606 | 44937833 |
| *KDM6A* | chrX | 44938266 | 44938497 |
| *KDM6A* | chrX | 44938448 | 44938676 |
| *KDM6A* | chrX | 44941692 | 44941929 |
| *KDM6A* | chrX | 44941870 | 44942125 |
| *KDM6A* | chrX | 44942674 | 44942932 |
| *KDM6A* | chrX | 44945069 | 44945323 |
| *KDM6A* | chrX | 44948859 | 44949090 |
| *KDM6A* | chrX | 44949035 | 44949297 |
| *KDM6A* | chrX | 44949929 | 44950201 |
| *KDM6A* | chrX | 44966624 | 44966848 |
| *KDM6A* | chrX | 44969285 | 44969533 |
| *KIT* | chr4 | 55589743 | 55590010 |
| *KIT* | chr4 | 55592111 | 55592355 |
| *KIT* | chr4 | 55593315 | 55593574 |
| *KIT* | chr4 | 55593517 | 55593777 |
| *KIT* | chr4 | 55599303 | 55599546 |
| *KRAS* | chr12 | 25380246 | 25380480 |
| *KRAS* | chr12 | 25398252 | 25398518 |
| *LUC7L2* | chr7 | 139044521 | 139044746 |
| *LUC7L2* | chr7 | 139044699 | 139044946 |
| *LUC7L2* | chr7 | 139044901 | 139045130 |
| *LUC7L2* | chr7 | 139060769 | 139061023 |
| *LUC7L2* | chr7 | 139083272 | 139083506 |
| *LUC7L2* | chr7 | 139086842 | 139087104 |
| *LUC7L2* | chr7 | 139090345 | 139090575 |
| *LUC7L2* | chr7 | 139091889 | 139092157 |
| *LUC7L2* | chr7 | 139094266 | 139094508 |
| *LUC7L2* | chr7 | 139097238 | 139097492 |
| *LUC7L2* | chr7 | 139102155 | 139102384 |
| *LUC7L2* | chr7 | 139102333 | 139102602 |
| *LUC7L2* | chr7 | 139106782 | 139107006 |
| *LUC7L2* | chr7 | 139106946 | 139107170 |
| *LUC7L2* | chr7 | 139107120 | 139107349 |
| *LUC7L2* | chr7 | 139107286 | 139107516 |
| *LUC7L2* | chr7 | 139107456 | 139107700 |
| *LUC7L2* | chr7 | 139107644 | 139107903 |
| *LUC7L2* | chr7 | 139107824 | 139108054 |
| *LUC7L2* | chr7 | 139108000 | 139108254 |
| *MECOM* | chr3 | 168801160 | 168801385 |
| *MECOM* | chr3 | 168801318 | 168801561 |
| *MECOM* | chr3 | 168801496 | 168801724 |
| *MECOM* | chr3 | 168801660 | 168801895 |
| *MECOM* | chr3 | 168801836 | 168802066 |
| *MECOM* | chr3 | 168802012 | 168802281 |
| *MECOM* | chr3 | 168802200 | 168802429 |
| *MECOM* | chr3 | 168802380 | 168802610 |
| *MECOM* | chr3 | 168802554 | 168802797 |
| *MECOM* | chr3 | 168802748 | 168803005 |
| *MECOM* | chr3 | 168806663 | 168806896 |
| *MECOM* | chr3 | 168806839 | 168807101 |
| *MECOM* | chr3 | 168807687 | 168807922 |
| *MECOM* | chr3 | 168807867 | 168808113 |
| *MECOM* | chr3 | 168810705 | 168810961 |
| *MECOM* | chr3 | 168812825 | 168813071 |
| *MECOM* | chr3 | 168818552 | 168818820 |
| *MECOM* | chr3 | 168819819 | 168820045 |
| *MECOM* | chr3 | 168825673 | 168825940 |
| *MECOM* | chr3 | 168830544 | 168830818 |
| *MECOM* | chr3 | 168833074 | 168833305 |
| *MECOM* | chr3 | 168833260 | 168833499 |
| *MECOM* | chr3 | 168833446 | 168833674 |
| *MECOM* | chr3 | 168833614 | 168833857 |
| *MECOM* | chr3 | 168833784 | 168834013 |
| *MECOM* | chr3 | 168833962 | 168834196 |
| *MECOM* | chr3 | 168834140 | 168834397 |
| *MECOM* | chr3 | 168834350 | 168834587 |
| *MECOM* | chr3 | 168838817 | 168839073 |
| *MECOM* | chr3 | 168840339 | 168840569 |
| *MECOM* | chr3 | 168845509 | 168845738 |
| *MECOM* | chr3 | 168845683 | 168845926 |
| *MECOM* | chr3 | 168849188 | 168849430 |
| *MECOM* | chr3 | 168861455 | 168861685 |
| *MECOM* | chr3 | 168862754 | 168863028 |
| *MECOM* | chr3 | 168863887 | 168864112 |
| *MECOM* | chr3 | 168864039 | 168864263 |
| *MECOM* | chr3 | 168864195 | 168864430 |
| *MECOM* | chr3 | 168864383 | 168864622 |
| *MECOM* | chr3 | 168864565 | 168864797 |
| *MECOM* | chr3 | 168864745 | 168864982 |
| *MECOM* | chr3 | 168865109 | 168865336 |
| *MECOM* | chr3 | 168865277 | 168865501 |
| *MECOM* | chr3 | 168865451 | 168865710 |
| *MECOM* | chr3 | 169098856 | 169099083 |
| *MECOM* | chr3 | 169099030 | 169099260 |
| *MECOM* | chr3 | 169099212 | 169099450 |
| *MECOM* | chr3 | 169381019 | 169381244 |
| *MECOM* | chr3 | 169381353 | 169381588 |
| *MPL* | chr1 | 43814903 | 43815132 |
| *NF1* | chr18 | 14156078 | 14156337 |
| *NF1* | chr18 | 14156283 | 14156508 |
| *NF1* | chr18 | 14156420 | 14156679 |
| *NF1* | chr18 | 14157032 | 14157274 |
| *NF1* | chr18 | 14157221 | 14157454 |
| *NF1* | chr21 | 15373135 | 15373368 |
| *NF1* | chr21 | 15373315 | 15373557 |
| *NF1* | chr21 | 15374079 | 15374304 |
| *NF1* | chr21 | 15374250 | 15374508 |
| *NF1* | chr22 | 16345883 | 16346115 |
| *NF1* | chr22 | 16346397 | 16346664 |
| *NF1* | chr22 | 16351949 | 16352172 |
| *NF1* | chr22 | 16352294 | 16352544 |
| *NF1* | chr14 | 19488785 | 19489036 |
| *NF1* | chr14 | 19489158 | 19489381 |
| *NF1* | chr14 | 19490983 | 19491213 |
| *NF1* | chr14 | 19494676 | 19494943 |
| *NF1* | chr14 | 19495225 | 19495457 |
| *NF1* | chr14 | 20078002 | 20078234 |
| *NF1* | chr14 | 20078516 | 20078783 |
| *NF1* | chr14 | 20082244 | 20082474 |
| *NF1* | chr14 | 20084076 | 20084299 |
| *NF1* | chr14 | 20084421 | 20084672 |
| *NF1* | chr14 | 20084954 | 20085207 |
| *NF1* | chr15 | 21125683 | 21125937 |
| *NF1* | chr15 | 21134282 | 21134502 |
| *NF1* | chr15 | 21134443 | 21134668 |
| *NF1* | chr15 | 21134609 | 21134834 |
| *NF1* | chr15 | 21136431 | 21136674 |
| *NF1* | chr15 | 21137026 | 21137284 |
| *NF1* | chr15 | 21137436 | 21137694 |
| *NF1* | chr15 | 21138153 | 21138385 |
| *NF1* | chr15 | 21138329 | 21138552 |
| *NF1* | chr15 | 21140130 | 21140360 |
| *NF1* | chr15 | 21140655 | 21140914 |
| *NF1* | chr15 | 22136864 | 22137118 |
| *NF1* | chr15 | 22145459 | 22145679 |
| *NF1* | chr15 | 22145620 | 22145845 |
| *NF1* | chr15 | 22145786 | 22146011 |
| *NF1* | chr15 | 22147608 | 22147851 |
| *NF1* | chr15 | 22148612 | 22148870 |
| *NF1* | chr15 | 22149329 | 22149561 |
| *NF1* | chr15 | 22149505 | 22149728 |
| *NF1* | chr15 | 22151308 | 22151538 |
| *NF1* | chr15 | 22151833 | 22152092 |
| *NF1* | chr17 | 29421822 | 29422047 |
| *NF1* | chr17 | 29421990 | 29422226 |
| *NF1* | chr17 | 29422176 | 29422443 |
| *NF1* | chr17 | 29482954 | 29483180 |
| *NF1* | chr17 | 29485983 | 29486227 |
| *NF1* | chr17 | 29490077 | 29490305 |
| *NF1* | chr17 | 29490255 | 29490528 |
| *NF1* | chr17 | 29496856 | 29497120 |
| *NF1* | chr17 | 29508313 | 29508562 |
| *NF1* | chr17 | 29508493 | 29508746 |
| *NF1* | chr17 | 29508691 | 29508945 |
| *NF1* | chr17 | 29509409 | 29509643 |
| *NF1* | chr17 | 29509589 | 29509847 |
| *NF1* | chr17 | 29527327 | 29527560 |
| *NF1* | chr17 | 29527507 | 29527749 |
| *NF1* | chr17 | 29527930 | 29528159 |
| *NF1* | chr17 | 29528102 | 29528363 |
| *NF1* | chr17 | 29528274 | 29528502 |
| *NF1* | chr17 | 29528448 | 29528706 |
| *NF1* | chr17 | 29533227 | 29533473 |
| *NF1* | chr17 | 29541438 | 29541670 |
| *NF1* | chr17 | 29545958 | 29546228 |
| *NF1* | chr17 | 29550431 | 29550697 |
| *NF1* | chr17 | 29552072 | 29552330 |
| *NF1* | chr17 | 29553552 | 29553813 |
| *NF1* | chr17 | 29554107 | 29554335 |
| *NF1* | chr17 | 29554285 | 29554519 |
| *NF1* | chr17 | 29554463 | 29554721 |
| *NF1* | chr17 | 29555940 | 29556164 |
| *NF1* | chr17 | 29556108 | 29556340 |
| *NF1* | chr17 | 29556286 | 29556532 |
| *NF1* | chr17 | 29556810 | 29557066 |
| *NF1* | chr17 | 29557223 | 29557481 |
| *NF1* | chr17 | 29557829 | 29558071 |
| *NF1* | chr17 | 29559036 | 29559310 |
| *NF1* | chr17 | 29559591 | 29559816 |
| *NF1* | chr17 | 29559757 | 29559982 |
| *NF1* | chr17 | 29559923 | 29560147 |
| *NF1* | chr17 | 29560093 | 29560355 |
| *NF1* | chr17 | 29562500 | 29562730 |
| *NF1* | chr17 | 29562674 | 29562917 |
| *NF1* | chr17 | 29562862 | 29563129 |
| *NF1* | chr17 | 29575873 | 29576126 |
| *NF1* | chr17 | 29576073 | 29576337 |
| *NF1* | chr17 | 29579927 | 29580167 |
| *NF1* | chr17 | 29585331 | 29585565 |
| *NF1* | chr17 | 29587356 | 29587625 |
| *NF1* | chr17 | 29588676 | 29588934 |
| *NF1* | chr17 | 29592214 | 29592446 |
| *NF1* | chr17 | 29652773 | 29653004 |
| *NF1* | chr17 | 29652947 | 29653187 |
| *NF1* | chr17 | 29653133 | 29653392 |
| *NF1* | chr17 | 29654390 | 29654614 |
| *NF1* | chr17 | 29654552 | 29654792 |
| *NF1* | chr17 | 29654740 | 29655001 |
| *NF1* | chr17 | 29657205 | 29657439 |
| *NF1* | chr17 | 29657383 | 29657611 |
| *NF1* | chr17 | 29661729 | 29661955 |
| *NF1* | chr17 | 29661901 | 29662146 |
| *NF1* | chr17 | 29663224 | 29663457 |
| *NF1* | chr17 | 29663396 | 29663660 |
| *NF1* | chr17 | 29663588 | 29663819 |
| *NF1* | chr17 | 29663768 | 29664039 |
| *NF1* | chr17 | 29664301 | 29664526 |
| *NF1* | chr17 | 29664481 | 29664751 |
| *NF1* | chr17 | 29664677 | 29664901 |
| *NF1* | chr17 | 29664831 | 29665063 |
| *NF1* | chr17 | 29665005 | 29665257 |
| *NF1* | chr17 | 29665651 | 29665885 |
| *NF1* | chr17 | 29667494 | 29667732 |
| *NF1* | chr17 | 29669994 | 29670266 |
| *NF1* | chr17 | 29676067 | 29676327 |
| *NF1* | chr17 | 29677174 | 29677431 |
| *NF1* | chr17 | 29679150 | 29679385 |
| *NF1* | chr17 | 29679340 | 29679575 |
| *NF1* | chr17 | 29683437 | 29683661 |
| *NF1* | chr17 | 29683873 | 29684100 |
| *NF1* | chr17 | 29684043 | 29684299 |
| *NF1* | chr17 | 29684243 | 29684499 |
| *NF1* | chr17 | 29685449 | 29685719 |
| *NF1* | chr17 | 29685948 | 29686215 |
| *NF1* | chr17 | 29687596 | 29687857 |
| *NF1* | chr17 | 29700906 | 29701130 |
| *NF1* | chr17 | 29701050 | 29701275 |
| *NF1* | chr17 | 29701192 | 29701416 |
| *NF1* | chr17 | 29701336 | 29701560 |
| *NF1* | chr17 | 29701504 | 29701728 |
| *NF1* | chr17 | 29701676 | 29701918 |
| *NF1* | chr17 | 29701860 | 29702126 |
| *NF1* | chr17 | 29702068 | 29702341 |
| *NF1* | chr17 | 29702268 | 29702498 |
| *NF1* | chr17 | 29702442 | 29702679 |
| *NF1* | chr17 | 29702624 | 29702857 |
| *NF1* | chr17 | 29702776 | 29703000 |
| *NF1* | chr17 | 29702940 | 29703164 |
| *NF1* | chr17 | 29703078 | 29703307 |
| *NF1* | chr17 | 29703220 | 29703444 |
| *NF1* | chr17 | 29703362 | 29703587 |
| *NF1* | chr17 | 29703530 | 29703755 |
| *NF1* | chr17 | 29703668 | 29703893 |
| *NF1* | chr17 | 29703830 | 29704056 |
| *NF1* | chr17 | 29704006 | 29704232 |
| *NF1* | chr17 | 29704150 | 29704384 |
| *NF1* | chr17 | 29704314 | 29704563 |
| *NF1* | chr17 | 29704508 | 29704742 |
| *NF1* | chr12 | 38599961 | 38600219 |
| *NF1* | chr12 | 38600503 | 38600753 |
| *NF1* | chr12 | 38600874 | 38601097 |
| *NF1* | chr2 | 131947881 | 131948162 |
| *NF1* | chr2 | 131948863 | 131949114 |
| *NF1* | chr2 | 131949236 | 131949459 |
| *NF1* | chr2 | 131951066 | 131951296 |
| *NF1* | chr2 | 131951563 | 131951820 |
| *NF1* | chr2 | 131953200 | 131953456 |
| *NF1* | chr2 | 131954831 | 131955097 |
| *NPM1* | chr2 | 4561631 | 4561900 |
| *NPM1* | chr12 | 9848721 | 9848990 |
| *NPM1* | chr7 | 11297147 | 11297414 |
| *NPM1* | chr7 | 18988361 | 18988627 |
| *NPM1* | chr10 | 21402325 | 21402593 |
| *NPM1* | chr13 | 28270364 | 28270633 |
| *NPM1* | chrX | 32224750 | 32225018 |
| *NPM1* | chr11 | 62099194 | 62099454 |
| *NPM1* | chr8 | 62115680 | 62115949 |
| *NPM1* | chr8 | 68098235 | 68098502 |
| *NPM1* | chr13 | 68405846 | 68406114 |
| *NPM1* | chr6 | 70415016 | 70415285 |
| *NPM1* | chr15 | 73453952 | 73454220 |
| *NPM1* | chr10 | 74677231 | 74677500 |
| *NPM1* | chr11 | 74790139 | 74790408 |
| *NPM1* | chr4 | 82931658 | 82931924 |
| *NPM1* | chr15 | 92829804 | 92830308 |
| *NPM1* | chr5 | 93018379 | 93018649 |
| *NPM1* | chr10 | 97948954 | 97949221 |
| *NPM1* | chr11 | 104552945 | 104553216 |
| *NPM1* | chrX | 123415169 | 123415438 |
| *NPM1* | chr3 | 147308631 | 147308882 |
| *NPM1* | chr7 | 149031845 | 149032109 |
| *NPM1* | chr5 | 170834711 | 170834971 |
| *NPM1* | chr5 | 170837487 | 170837733 |
| *NPM1* | chr2 | 198244262 | 198244531 |
| *NPM1* | chr2 | 204637191 | 204637460 |
| *NRAS* | chr1 | 115258716 | 115258982 |
| *PHF6* | chrX | 133507313 | 133507553 |
| *PHF6* | chrX | 133511509 | 133511769 |
| *PHF6* | chrX | 133511715 | 133511989 |
| *PHF6* | chrX | 133511925 | 133512163 |
| *PHF6* | chrX | 133527416 | 133527666 |
| *PHF6* | chrX | 133527610 | 133527884 |
| *PHF6* | chrX | 133527830 | 133528098 |
| *PHF6* | chrX | 133547397 | 133547670 |
| *PHF6* | chrX | 133547617 | 133547889 |
| *PHF6* | chrX | 133547833 | 133548073 |
| *PHF6* | chrX | 133548933 | 133549177 |
| *PHF6* | chrX | 133549121 | 133549394 |
| *PHF6* | chrX | 133551168 | 133551428 |
| *PHF6* | chrX | 133559108 | 133559332 |
| *PHF6* | chrX | 133559252 | 133559485 |
| *PHF6* | chrX | 133559398 | 133559622 |
| *PHF6* | chrX | 133559556 | 133559782 |
| *PHF6* | chrX | 133559698 | 133559923 |
| *PHF6* | chrX | 133559838 | 133560064 |
| *PHF6* | chrX | 133559978 | 133560204 |
| *PHF6* | chrX | 133560126 | 133560352 |
| *PHF6* | chrX | 133560270 | 133560496 |
| *PHF6* | chrX | 133560414 | 133560639 |
| *PHF6* | chrX | 133560552 | 133560776 |
| *PHF6* | chrX | 133560692 | 133560917 |
| *PHF6* | chrX | 133560838 | 133561065 |
| *PHF6* | chrX | 133561008 | 133561233 |
| *PHF6* | chrX | 133561146 | 133561371 |
| *PHF6* | chrX | 133561286 | 133561512 |
| *PHF6* | chrX | 133561448 | 133561678 |
| *PHF6* | chrX | 133561622 | 133561846 |
| *PHF6* | chrX | 133561788 | 133562021 |
| *PHF6* | chrX | 133561966 | 133562237 |
| *PHF6* | chrX | 133562178 | 133562411 |
| *PHF6* | chrX | 133562350 | 133562612 |
| *PHF6* | chrX | 133562532 | 133562783 |
| *PHF6* | chrX | 133562724 | 133562990 |
| *PRPF8* | chr17 | 1553806 | 1554032 |
| *PRPF8* | chr17 | 1553958 | 1554227 |
| *PRPF8* | chr17 | 1554176 | 1554447 |
| *PRPF8* | chr17 | 1554400 | 1554626 |
| *PRPF8* | chr17 | 1554554 | 1554783 |
| *PRPF8* | chr17 | 1554734 | 1554964 |
| *PRPF8* | chr17 | 1554916 | 1555156 |
| *PRPF8* | chr17 | 1556707 | 1556940 |
| *PRPF8* | chr17 | 1556863 | 1557094 |
| *PRPF8* | chr17 | 1557045 | 1557288 |
| *PRPF8* | chr17 | 1557235 | 1557495 |
| *PRPF8* | chr17 | 1558617 | 1558869 |
| *PRPF8* | chr17 | 1559561 | 1559790 |
| *PRPF8* | chr17 | 1559737 | 1559966 |
| *PRPF8* | chr17 | 1559921 | 1560183 |
| *PRPF8* | chr17 | 1561422 | 1561651 |
| *PRPF8* | chr17 | 1561818 | 1562048 |
| *PRPF8* | chr17 | 1561996 | 1562233 |
| *PRPF8* | chr17 | 1562622 | 1562872 |
| *PRPF8* | chr17 | 1563108 | 1563338 |
| *PRPF8* | chr17 | 1563601 | 1563827 |
| *PRPF8* | chr17 | 1563745 | 1563971 |
| *PRPF8* | chr17 | 1563887 | 1564117 |
| *PRPF8* | chr17 | 1564063 | 1564336 |
| *PRPF8* | chr17 | 1564285 | 1564510 |
| *PRPF8* | chr17 | 1564433 | 1564659 |
| *PRPF8* | chr17 | 1564605 | 1564831 |
| *PRPF8* | chr17 | 1564777 | 1565008 |
| *PRPF8* | chr17 | 1564955 | 1565190 |
| *PRPF8* | chr17 | 1565131 | 1565355 |
| *PRPF8* | chr17 | 1565301 | 1565564 |
| *PRPF8* | chr17 | 1576250 | 1576476 |
| *PRPF8* | chr17 | 1576428 | 1576658 |
| *PRPF8* | chr17 | 1576596 | 1576822 |
| *PRPF8* | chr17 | 1576776 | 1577012 |
| *PRPF8* | chr17 | 1576964 | 1577237 |
| *PRPF8* | chr17 | 1577611 | 1577846 |
| *PRPF8* | chr17 | 1577797 | 1578059 |
| *PRPF8* | chr17 | 1578415 | 1578685 |
| *PRPF8* | chr17 | 1578787 | 1579013 |
| *PRPF8* | chr17 | 1578931 | 1579155 |
| *PRPF8* | chr17 | 1579091 | 1579316 |
| *PRPF8* | chr17 | 1579267 | 1579492 |
| *PRPF8* | chr17 | 1579443 | 1579670 |
| *PRPF8* | chr17 | 1579619 | 1579843 |
| *PRPF8* | chr17 | 1579795 | 1580020 |
| *PRPF8* | chr17 | 1579957 | 1580226 |
| *PRPF8* | chr17 | 1580173 | 1580403 |
| *PRPF8* | chr17 | 1580349 | 1580596 |
| *PRPF8* | chr17 | 1580830 | 1581080 |
| *PRPF8* | chr17 | 1581841 | 1582065 |
| *PRPF8* | chr17 | 1581993 | 1582220 |
| *PRPF8* | chr17 | 1582169 | 1582395 |
| *PRPF8* | chr17 | 1582343 | 1582570 |
| *PRPF8* | chr17 | 1582519 | 1582747 |
| *PRPF8* | chr17 | 1582697 | 1582927 |
| *PRPF8* | chr17 | 1582877 | 1583149 |
| *PRPF8* | chr17 | 1583897 | 1584126 |
| *PRPF8* | chr17 | 1584071 | 1584295 |
| *PRPF8* | chr17 | 1584241 | 1584505 |
| *PRPF8* | chr17 | 1584659 | 1584884 |
| *PRPF8* | chr17 | 1584811 | 1585059 |
| *PRPF8* | chr17 | 1585007 | 1585243 |
| *PRPF8* | chr17 | 1585191 | 1585439 |
| *PRPF8* | chr17 | 1585389 | 1585629 |
| *PRPF8* | chr17 | 1586796 | 1587061 |
| *PRPF8* | chr17 | 1587639 | 1587867 |
| *PRPF8* | chr17 | 1587803 | 1588035 |
| *PRPF8* | chr17 | 1587989 | 1588226 |
| *PTPN11* | chr12 | 112888162 | 112888418 |
| *PTPN11* | chr12 | 112926856 | 112927108 |
| *RAD21* | chr12 | 8632388 | 8632615 |
| *RAD21* | chrX | 100057976 | 100058219 |
| *RAD21* | chrX | 100058339 | 100058565 |
| *RAD21* | chrX | 100058678 | 100058914 |
| *RAD21* | chrX | 100059031 | 100059555 |
| *RAD21* | chrX | 100059197 | 100059414 |
| *RAD21* | chr8 | 117858054 | 117858284 |
| *RAD21* | chr8 | 117858210 | 117858436 |
| *RAD21* | chr8 | 117858352 | 117858576 |
| *RAD21* | chr8 | 117858504 | 117858742 |
| *RAD21* | chr8 | 117858684 | 117858910 |
| *RAD21* | chr8 | 117858858 | 117859095 |
| *RAD21* | chr8 | 117859038 | 117859266 |
| *RAD21* | chr8 | 117859212 | 117859444 |
| *RAD21* | chr8 | 117859388 | 117859628 |
| *RAD21* | chr8 | 117859570 | 117859814 |
| *RAD21* | chr8 | 117859764 | 117859996 |
| *RAD21* | chr8 | 117861158 | 117861426 |
| *RAD21* | chr8 | 117862814 | 117863088 |
| *RAD21* | chr8 | 117864154 | 117864386 |
| *RAD21* | chr8 | 117864757 | 117865031 |
| *RAD21* | chr8 | 117866363 | 117866590 |
| *RAD21* | chr8 | 117866537 | 117866794 |
| *RAD21* | chr8 | 117868374 | 117868610 |
| *RAD21* | chr8 | 117868856 | 117869095 |
| *RAD21* | chr8 | 117869401 | 117869631 |
| *RAD21* | chr8 | 117869575 | 117869823 |
| *RAD21* | chr8 | 117870526 | 117870767 |
| *RAD21* | chr8 | 117874033 | 117874301 |
| *RAD21* | chr8 | 117875338 | 117875610 |
| *RAD21* | chr8 | 117878770 | 117879007 |
| *RAD21* | chr8 | 117878950 | 117879220 |
| *RAD21* | chr8 | 117886751 | 117887024 |
| *RAD21* | chr8 | 117886971 | 117887212 |
| *RUNX1* | chr21 | 36159971 | 36160195 |
| *RUNX1* | chr21 | 36160113 | 36160338 |
| *RUNX1* | chr21 | 36160259 | 36160483 |
| *RUNX1* | chr21 | 36160407 | 36160636 |
| *RUNX1* | chr21 | 36160573 | 36160827 |
| *RUNX1* | chr21 | 36160741 | 36160989 |
| *RUNX1* | chr21 | 36160921 | 36161146 |
| *RUNX1* | chr21 | 36161067 | 36161292 |
| *RUNX1* | chr21 | 36161207 | 36161431 |
| *RUNX1* | chr21 | 36161353 | 36161578 |
| *RUNX1* | chr21 | 36161495 | 36161720 |
| *RUNX1* | chr21 | 36161633 | 36161866 |
| *RUNX1* | chr21 | 36161787 | 36162011 |
| *RUNX1* | chr21 | 36161927 | 36162151 |
| *RUNX1* | chr21 | 36162065 | 36162291 |
| *RUNX1* | chr21 | 36162211 | 36162435 |
| *RUNX1* | chr21 | 36162349 | 36162574 |
| *RUNX1* | chr21 | 36162489 | 36162716 |
| *RUNX1* | chr21 | 36162627 | 36162851 |
| *RUNX1* | chr21 | 36162765 | 36162989 |
| *RUNX1* | chr21 | 36162909 | 36163134 |
| *RUNX1* | chr21 | 36163053 | 36163278 |
| *RUNX1* | chr21 | 36163191 | 36163416 |
| *RUNX1* | chr21 | 36163335 | 36163559 |
| *RUNX1* | chr21 | 36163479 | 36163703 |
| *RUNX1* | chr21 | 36163645 | 36163904 |
| *RUNX1* | chr21 | 36163833 | 36164090 |
| *RUNX1* | chr21 | 36164361 | 36164586 |
| *RUNX1* | chr21 | 36164535 | 36164764 |
| *RUNX1* | chr21 | 36164711 | 36164952 |
| *RUNX1* | chr21 | 36171569 | 36171803 |
| *RUNX1* | chr21 | 36193453 | 36193678 |
| *RUNX1* | chr21 | 36193619 | 36193852 |
| *RUNX1* | chr21 | 36193799 | 36194041 |
| *RUNX1* | chr21 | 36206584 | 36206813 |
| *RUNX1* | chr21 | 36206764 | 36207021 |
| *RUNX1* | chr21 | 36231744 | 36232002 |
| *RUNX1* | chr21 | 36252823 | 36253071 |
| *RUNX1* | chr21 | 36259017 | 36259242 |
| *RUNX1* | chr21 | 36259341 | 36259568 |
| *RUNX1* | chr21 | 36259503 | 36259761 |
| *RUNX1* | chr21 | 36259705 | 36259933 |
| *RUNX1* | chr21 | 36259877 | 36260123 |
| *RUNX1* | chr21 | 36260061 | 36260285 |
| *RUNX1* | chr21 | 36260227 | 36260452 |
| *RUNX1* | chr21 | 36260403 | 36260628 |
| *RUNX1* | chr21 | 36260579 | 36260843 |
| *RUNX1* | chr21 | 36260765 | 36261029 |
| *RUNX1* | chr21 | 36260977 | 36261238 |
| *RUNX1* | chr21 | 36265167 | 36265391 |
| *RUNX1* | chr21 | 36421020 | 36421255 |
| *RUNX1* | chr21 | 36421202 | 36421476 |
| *RUNX1* | chr21 | 36421428 | 36421664 |
| *SETBP1* | chr18 | 42531804 | 42532043 |
| *SF3B1* | chr2 | 198266684 | 198266908 |
| *SF3B1* | chr2 | 198267329 | 198267565 |
| *SMC3* | chr2 | 99719054 | 99719437 |
| *SMC3* | chr10 | 112327412 | 112327644 |
| *SMC3* | chr10 | 112328659 | 112328933 |
| *SMC3* | chr10 | 112335019 | 112335275 |
| *SMC3* | chr10 | 112337148 | 112337422 |
| *SMC3* | chr10 | 112337510 | 112337742 |
| *SMC3* | chr10 | 112338335 | 112338609 |
| *SMC3* | chr10 | 112340621 | 112340867 |
| *SMC3* | chr10 | 112341652 | 112341926 |
| *SMC3* | chr10 | 112342203 | 112342455 |
| *SMC3* | chr10 | 112343089 | 112343363 |
| *SMC3* | chr10 | 112343478 | 112343708 |
| *SMC3* | chr10 | 112343654 | 112343881 |
| *SMC3* | chr10 | 112343820 | 112344052 |
| *SMC3* | chr10 | 112343998 | 112344259 |
| *SMC3* | chr10 | 112349236 | 112349503 |
| *SMC3* | chr10 | 112349436 | 112349692 |
| *SMC3* | chr10 | 112349642 | 112349866 |
| *SMC3* | chr10 | 112350041 | 112350277 |
| *SMC3* | chr10 | 112350221 | 112350467 |
| *SMC3* | chr10 | 112350710 | 112350972 |
| *SMC3* | chr10 | 112352800 | 112353057 |
| *SMC3* | chr10 | 112356121 | 112356359 |
| *SMC3* | chr10 | 112357864 | 112358089 |
| *SMC3* | chr10 | 112359371 | 112359645 |
| *SMC3* | chr10 | 112360154 | 112360398 |
| *SMC3* | chr10 | 112360735 | 112360981 |
| *SMC3* | chr10 | 112361266 | 112361491 |
| *SMC3* | chr10 | 112361408 | 112361651 |
| *SMC3* | chr10 | 112361598 | 112361828 |
| *SMC3* | chr10 | 112361772 | 112362027 |
| *SMC3* | chr10 | 112362105 | 112362330 |
| *SMC3* | chr10 | 112362269 | 112362502 |
| *SMC3* | chr10 | 112362443 | 112362672 |
| *SMC3* | chr10 | 112362619 | 112362857 |
| *SMC3* | chr10 | 112362781 | 112363012 |
| *SMC3* | chr10 | 112362959 | 112363226 |
| *SMC3* | chr10 | 112363886 | 112364124 |
| *SMC3* | chr10 | 112364056 | 112364283 |
| *SMC3* | chr10 | 112364222 | 112364477 |
| *SMC3* | chr7 | 131246446 | 131246832 |
| *STAG2* | chr2 | 98290091 | 98290572 |
| *STAG2* | chr10 | 122931324 | 122931501 |
| *STAG2* | chrX | 123094350 | 123094592 |
| *STAG2* | chrX | 123094540 | 123094765 |
| *STAG2* | chrX | 123095517 | 123095745 |
| *STAG2* | chrX | 123155184 | 123155430 |
| *STAG2* | chrX | 123156344 | 123156614 |
| *STAG2* | chrX | 123159659 | 123159883 |
| *STAG2* | chrX | 123164684 | 123164909 |
| *STAG2* | chrX | 123164856 | 123165125 |
| *STAG2* | chrX | 123171302 | 123171528 |
| *STAG2* | chrX | 123176346 | 123176592 |
| *STAG2* | chrX | 123178887 | 123179154 |
| *STAG2* | chrX | 123179097 | 123179358 |
| *STAG2* | chrX | 123181163 | 123181403 |
| *STAG2* | chrX | 123182816 | 123183070 |
| *STAG2* | chrX | 123183997 | 123184261 |
| *STAG2* | chrX | 123184844 | 123185112 |
| *STAG2* | chrX | 123185056 | 123185327 |
| *STAG2* | chrX | 123189947 | 123190221 |
| *STAG2* | chrX | 123191611 | 123191883 |
| *STAG2* | chrX | 123195043 | 123195315 |
| *STAG2* | chrX | 123195570 | 123195820 |
| *STAG2* | chrX | 123196623 | 123196850 |
| *STAG2* | chrX | 123196785 | 123197014 |
| *STAG2* | chrX | 123196961 | 123197204 |
| *STAG2* | chrX | 123197571 | 123197800 |
| *STAG2* | chrX | 123197745 | 123198002 |
| *STAG2* | chrX | 123199597 | 123199821 |
| *STAG2* | chrX | 123199739 | 123199964 |
| *STAG2* | chrX | 123199881 | 123200108 |
| *STAG2* | chrX | 123200053 | 123200277 |
| *STAG2* | chrX | 123200221 | 123200463 |
| *STAG2* | chrX | 123202343 | 123202615 |
| *STAG2* | chrX | 123204956 | 123205226 |
| *STAG2* | chrX | 123210153 | 123210403 |
| *STAG2* | chrX | 123211754 | 123212004 |
| *STAG2* | chrX | 123215199 | 123215463 |
| *STAG2* | chrX | 123217142 | 123217390 |
| *STAG2* | chrX | 123217336 | 123217609 |
| *STAG2* | chrX | 123220270 | 123220501 |
| *STAG2* | chrX | 123220452 | 123220698 |
| *STAG2* | chrX | 123224300 | 123224527 |
| *STAG2* | chrX | 123224474 | 123224731 |
| *STAG2* | chrX | 123224676 | 123224930 |
| *STAG2* | chrX | 123227827 | 123228093 |
| *STAG2* | chrX | 123229183 | 123229427 |
| *STAG2* | chrX | 123234295 | 123234527 |
| *STAG2* | chrX | 123234469 | 123234702 |
| *STAG2* | chrX | 123234633 | 123234894 |
| *STAG2* | chrX | 123234837 | 123235111 |
| *STAG2* | chrX | 123235053 | 123235278 |
| *STAG2* | chrX | 123235199 | 123235428 |
| *STAG2* | chrX | 123235343 | 123235571 |
| *STAG2* | chrX | 123235513 | 123235745 |
| *STAG2* | chrX | 123235691 | 123235928 |
| *STAG2* | chrX | 123235869 | 123236098 |
| *STAG2* | chrX | 123236037 | 123236262 |
| *STAG2* | chrX | 123236189 | 123236414 |
| *STAG2* | chrX | 123236357 | 123236622 |
| *STAT3* | chr17 | 40475025 | 40475272 |
| *STAT3* | chr17 | 40475221 | 40475461 |
| *SUZ12* | chr17 | 29058574 | 29058844 |
| *SUZ12* | chr17 | 29058799 | 29059050 |
| *SUZ12* | chr17 | 29059001 | 29059239 |
| *SUZ12* | chr17 | 29061994 | 29062263 |
| *SUZ12* | chr17 | 29070278 | 29070514 |
| *SUZ12* | chr17 | 29086454 | 29086711 |
| *SUZ12* | chr17 | 29093452 | 29093678 |
| *SUZ12* | chr17 | 29095723 | 29095950 |
| *SUZ12* | chr17 | 29095899 | 29096158 |
| *SUZ12* | chr17 | 29096823 | 29097047 |
| *SUZ12* | chr17 | 29103280 | 29103514 |
| *SUZ12* | chr17 | 30263935 | 30264206 |
| *SUZ12* | chr17 | 30264161 | 30264424 |
| *SUZ12* | chr17 | 30264375 | 30264637 |
| *SUZ12* | chr17 | 30267178 | 30267443 |
| *SUZ12* | chr17 | 30267384 | 30267657 |
| *SUZ12* | chr17 | 30274595 | 30274831 |
| *SUZ12* | chr17 | 30293109 | 30293367 |
| *SUZ12* | chr17 | 30300096 | 30300322 |
| *SUZ12* | chr17 | 30302372 | 30302599 |
| *SUZ12* | chr17 | 30302548 | 30302808 |
| *SUZ12* | chr17 | 30303473 | 30303697 |
| *SUZ12* | chr17 | 30309983 | 30310217 |
| *SUZ12* | chr17 | 30315304 | 30315577 |
| *SUZ12* | chr17 | 30320142 | 30320372 |
| *SUZ12* | chr17 | 30320314 | 30320560 |
| *SUZ12* | chr17 | 30320839 | 30321081 |
| *SUZ12* | chr17 | 30321458 | 30321700 |
| *SUZ12* | chr17 | 30321648 | 30321905 |
| *SUZ12* | chr17 | 30322460 | 30322699 |
| *SUZ12* | chr17 | 30322642 | 30322877 |
| *SUZ12* | chr17 | 30323786 | 30324042 |
| *SUZ12* | chr17 | 30325596 | 30325836 |
| *SUZ12* | chr17 | 30325778 | 30326006 |
| *SUZ12* | chr17 | 30325952 | 30326178 |
| *SUZ12* | chr17 | 30326122 | 30326372 |
| *SUZ12* | chr17 | 30326286 | 30326533 |
| *SUZ12* | chr17 | 30326448 | 30326688 |
| *SUZ12* | chr17 | 30326630 | 30326873 |
| *SUZ12* | chr17 | 30326812 | 30327041 |
| *SUZ12* | chr17 | 30326980 | 30327253 |
| *SUZ12* | chr17 | 30327168 | 30327429 |
| *SUZ12* | chr17 | 30327368 | 30327593 |
| *SUZ12* | chr17 | 30327536 | 30327760 |
| *SUZ12* | chr17 | 30327702 | 30327932 |
| *SUZ12* | chr17 | 30327876 | 30328147 |
| *TET2* | chr6 | 3847213 | 3847441 |
| *TET2* | chr3 | 5871749 | 5871959 |
| *TET2* | chr20 | 7430743 | 7430971 |
| *TET2* | chr4 | 17676983 | 17677190 |
| *TET2* | chr7 | 18587336 | 18587747 |
| *TET2* | chr21 | 23677856 | 23678082 |
| *TET2* | chr16 | 25681182 | 25681405 |
| *TET2* | chr14 | 31802840 | 31803353 |
| *TET2* | chr9 | 31813242 | 31813468 |
| *TET2* | chrX | 33340391 | 33340622 |
| *TET2* | chr12 | 33600790 | 33601017 |
| *TET2* | chr18 | 42986805 | 42987032 |
| *TET2* | chrX | 50630067 | 50630284 |
| *TET2* | chr7 | 55520298 | 55520498 |
| *TET2* | chr3 | 57672384 | 57672612 |
| *TET2* | chr5 | 57732615 | 57732843 |
| *TET2* | chr5 | 59272699 | 59272926 |
| *TET2* | chr8 | 70880182 | 70880421 |
| *TET2* | chr2 | 72175292 | 72175506 |
| *TET2* | chr8 | 72361723 | 72361952 |
| *TET2* | chr3 | 74445091 | 74445335 |
| *TET2* | chr13 | 85769283 | 85769506 |
| *TET2* | chr6 | 89659690 | 89659909 |
| *TET2* | chr8 | 93327960 | 93328189 |
| *TET2* | chr1 | 96591803 | 96592015 |
| *TET2* | chr8 | 97037591 | 97037821 |
| *TET2* | chr4 | 99522222 | 99522433 |
| *TET2* | chr4 | 106067916 | 106068185 |
| *TET2* | chr4 | 106111486 | 106111748 |
| *TET2* | chr4 | 106154927 | 106155152 |
| *TET2* | chr4 | 106155075 | 106155300 |
| *TET2* | chr4 | 106155215 | 106155439 |
| *TET2* | chr4 | 106155359 | 106155583 |
| *TET2* | chr4 | 106155505 | 106155729 |
| *TET2* | chr4 | 106155649 | 106155874 |
| *TET2* | chr4 | 106155787 | 106156011 |
| *TET2* | chr4 | 106155929 | 106156153 |
| *TET2* | chr4 | 106156067 | 106156291 |
| *TET2* | chr4 | 106156207 | 106156432 |
| *TET2* | chr4 | 106156351 | 106156576 |
| *TET2* | chr4 | 106156493 | 106156717 |
| *TET2* | chr4 | 106156635 | 106156859 |
| *TET2* | chr4 | 106156775 | 106157000 |
| *TET2* | chr4 | 106156937 | 106157161 |
| *TET2* | chr4 | 106157081 | 106157305 |
| *TET2* | chr4 | 106157221 | 106157446 |
| *TET2* | chr4 | 106157513 | 106157737 |
| *TET2* | chr4 | 106157653 | 106157878 |
| *TET2* | chr4 | 106157795 | 106158022 |
| *TET2* | chr4 | 106157943 | 106158167 |
| *TET2* | chr4 | 106158087 | 106158311 |
| *TET2* | chr4 | 106158253 | 106158478 |
| *TET2* | chr4 | 106158389 | 106158617 |
| *TET2* | chr4 | 106158539 | 106158765 |
| *TET2* | chr4 | 106158683 | 106158907 |
| *TET2* | chr4 | 106158823 | 106159048 |
| *TET2* | chr4 | 106158969 | 106159214 |
| *TET2* | chr4 | 106159153 | 106159400 |
| *TET2* | chr4 | 106159343 | 106159567 |
| *TET2* | chr4 | 106159483 | 106159708 |
| *TET2* | chr4 | 106159625 | 106159853 |
| *TET2* | chr4 | 106159771 | 106159997 |
| *TET2* | chr4 | 106159921 | 106160148 |
| *TET2* | chr4 | 106160089 | 106160320 |
| *TET2* | chr4 | 106160251 | 106160477 |
| *TET2* | chr4 | 106160857 | 106161086 |
| *TET2* | chr4 | 106161019 | 106161254 |
| *TET2* | chr4 | 106161185 | 106161441 |
| *TET2* | chr4 | 106161385 | 106161610 |
| *TET2* | chr4 | 106161553 | 106161782 |
| *TET2* | chr4 | 106161731 | 106161968 |
| *TET2* | chr4 | 106161901 | 106162125 |
| *TET2* | chr4 | 106162063 | 106162336 |
| *TET2* | chr4 | 106162275 | 106162544 |
| *TET2* | chr4 | 106162485 | 106162719 |
| *TET2* | chr4 | 106162641 | 106162877 |
| *TET2* | chr4 | 106162819 | 106163048 |
| *TET2* | chr4 | 106162977 | 106163201 |
| *TET2* | chr4 | 106163147 | 106163372 |
| *TET2* | chr4 | 106163305 | 106163530 |
| *TET2* | chr4 | 106163469 | 106163719 |
| *TET2* | chr4 | 106163663 | 106163888 |
| *TET2* | chr4 | 106163823 | 106164051 |
| *TET2* | chr4 | 106163997 | 106164251 |
| *TET2* | chr4 | 106164600 | 106164838 |
| *TET2* | chr4 | 106164784 | 106165026 |
| *TET2* | chr4 | 106180745 | 106181005 |
| *TET2* | chr4 | 106182853 | 106183085 |
| *TET2* | chr4 | 106190740 | 106190993 |
| *TET2* | chr4 | 106193596 | 106193822 |
| *TET2* | chr4 | 106193764 | 106194021 |
| *TET2* | chrX | 113733329 | 113733552 |
| *TET2* | chr4 | 130405906 | 130406104 |
| *TET2* | chr4 | 131884887 | 131885113 |
| *TET2* | chr6 | 149737559 | 149737784 |
| *TET2* | chr2 | 150042376 | 150042602 |
| *TET2* | chr6 | 150875148 | 150875371 |
| *TET2* | chr7 | 154495729 | 154495956 |
| *TET2* | chr5 | 157272936 | 157273146 |
| *TET2* | chr3 | 164560378 | 164560593 |
| *TET2* | chr4 | 166845929 | 166846155 |
| *TET2* | chr2 | 170525082 | 170525310 |
| *TET2* | chr1 | 191455791 | 191456020 |
| *TET2* | chr1 | 228687327 | 228687764 |
| *TP53* | chr17 | 7571623 | 7571854 |
| *TP53* | chr17 | 7572181 | 7572405 |
| *TP53* | chr17 | 7572345 | 7572575 |
| *TP53* | chr17 | 7572519 | 7572753 |
| *TP53* | chr17 | 7572701 | 7572953 |
| *TP53* | chr17 | 7572901 | 7573127 |
| *TP53* | chr17 | 7573904 | 7574178 |
| *TP53* | chr17 | 7576588 | 7576826 |
| *TP53* | chr17 | 7576772 | 7577005 |
| *TP53* | chr17 | 7576956 | 7577194 |
| *TP53* | chr17 | 7578052 | 7578281 |
| *TP53* | chr17 | 7578230 | 7578455 |
| *TP53* | chr17 | 7578410 | 7578634 |
| *TP53* | chr17 | 7590666 | 7590928 |
| *U2AF1* | chr21 | 44514750 | 44515010 |
| *U2AF1* | chr21 | 44524429 | 44524703 |
| *WT1* | chr11 | 32409197 | 32409441 |
| *WT1* | chr11 | 32409385 | 32409658 |
| *WT1* | chr11 | 32409585 | 32409821 |
| *WT1* | chr11 | 32409765 | 32409993 |
| *WT1* | chr11 | 32409921 | 32410185 |
| *WT1* | chr11 | 32410129 | 32410353 |
| *WT1* | chr11 | 32410299 | 32410533 |
| *WT1* | chr11 | 32410477 | 32410706 |
| *WT1* | chr11 | 32410657 | 32410895 |
| *WT1* | chr11 | 32413487 | 32413749 |
| *WT1* | chr11 | 32417778 | 32418052 |
| *WT1* | chr11 | 32421467 | 32421723 |
| *WT1* | chr11 | 32438007 | 32438236 |
| *WT1* | chr11 | 32439092 | 32439340 |
| *WT1* | chr11 | 32449479 | 32449710 |
| *WT1* | chr11 | 32450016 | 32450272 |
| *WT1* | chr11 | 32451957 | 32452184 |
| *WT1* | chr11 | 32452135 | 32452388 |
| *WT1* | chr11 | 32456121 | 32456346 |
| *WT1* | chr11 | 32456291 | 32456547 |
| *WT1* | chr11 | 32456659 | 32456884 |
| *WT1* | chr11 | 32456839 | 32457068 |
| *WT1* | chr11 | 32457015 | 32457286 |
| *ZRSR2* | chrX | 15808545 | 15808778 |
| *ZRSR2* | chrX | 15809026 | 15809262 |
| *ZRSR2* | chrX | 15817958 | 15818232 |
| *ZRSR2* | chrX | 15821772 | 15821998 |
| *ZRSR2* | chrX | 15822203 | 15822473 |
| *ZRSR2* | chrX | 15826325 | 15826596 |
| *ZRSR2* | chrX | 15827292 | 15827558 |
| *ZRSR2* | chrX | 15836641 | 15836866 |
| *ZRSR2* | chrX | 15838297 | 15838533 |
| *ZRSR2* | chrX | 15840735 | 15840965 |
| *ZRSR2* | chrX | 15840907 | 15841133 |
| *ZRSR2* | chrX | 15841077 | 15841309 |
| *ZRSR2* | chr5 | 112228512 | 112228720 |
